# Chicago Dental Club

**Published:** 1890-06

**Authors:** C. Stoddard Smith

**Affiliations:** Secretary


### Reports of Society Meetings.

### CHICAGO DENTAL CLUB.[1]

The monthly meeting of the Chicago Dental Club was held on Monday evening, March 24, 1890, the President, Dr. A. E. Matteson, in the chair.

Drs. J. H. Martin, J. W. Slonaker, Marvin E. Smith, and M. A. Newman were elected members, and, after some miscellaneous and routine business, the subject of the evening was opened by the reading of a paper, by Dr. C. Stoddard Smith, on "Dental Education." (For paper, see page 339).

The subject being declared open for discussion, Dr. E. L. Clifford said,—

Mr. President and Gentlemen,—It has been our privilege, this evening, to listen to one of the most practical, most substantial, and, I may say, one of the most instructive papers that has been placed on the programme of our monthly meetings for some time. It is not necessary to say interesting, in speaking of such an effort upon such a subject, for does it not constitute the very foundation of our professional standing and progress, and would it be possible to conceive of "a man with soul so dead" in our rapidly-increasing roll of membership that could not, or would not, appreciate the importance of full and free discussion upon such a topic.

Discussion! May I be pardoned for digressing just one moment, to allude to the true meaning and importance of discussion. Usage adds to our programme the discussion of papers read before our club, and by discussion, I take it, the fact is not meant that your time and mine shall be taxed to listen to deserved or undeserved eulogy, praise, and compliment. Our papers are presented to us for mutual, scientific instruction, and the discussion upon these

---

[1] Reported for the International Dental Journal, by C. Stoddard Smith, D.D.S.

papers is for the purpose of giving to the club the individual ideas of its membership upon the subjects presented. Necessarily, such discussions may be critical, but I ask you to bear in mind that criticism, pure and simple, is not fault finding or blaming. It is a privilege accorded the humblest member of this club to express his views and opinions upon any paper, and should these opinions or remarks differ or agree with the statements of any essayist, the reception should be the same and the speaker accredited with at least being conscientious. Though we work, and even fight, for the promulgation of such ideas as we deem correct, let it be determined that, when the portals of our club-room close behind us, the mantle of charity shall enshrine and embrace each individual member.

Should I, therefore, see fit to exercise my prerogative of taking issue with some of the points made by the essayist, do me the justice of ascribing honesty of motive.

The essayist gives us, in brief, a history of the advancement of dental education. He takes us back to the time when all knowledge, all education in our specialty, was practical; when the embryonic state of our profession did not call for theory, and all the advantages which he has depicted as arising from a theoretical training, and leads us step by step through what might be termed the seven stages of man's development. With this idea, in the abstract, that manual, practical training is all-important and essential, I would not ask to differ. Apprenticeships I believe to be absolutely necessary, and only regret that, in addition to the preliminary examinations required by the colleges, one year's tutelage in an office is not also obligatory. It is true, as the essayist says, that if we take a greenhorn from the average walks of life and place him under the task of imbibing from didactic lectures, much time must necessarily be lost in his trying to familiarize himself with the technicalities of his selection. In this, I believe, I can speak from experience, having watched the comparative advancement of such students in the college. The essayist suggests that the object of dental education is to make dentists. With this suggestion I am in full accord, but acknowledge the fact and must claim the belief that the dental office should be the starting-point for practical training. No man can appreciate more than I do the value of office tutelage. When I entered college for the purpose of gaining the finishing touches to a professional life, had I been compelled to depend upon the practical training there received (and the advantages were as good as the best), I certainly think I should still be working for the longed-for diploma. But the question arises, What prompted me to covet this diploma and to attend college? The fact that my profession had advanced to that state where theory and theoretical training was absolutely essential. Now, what caused this advancement? The realization by the older heads and brightest lights of dentistry that our specialty was no less than a branch of the healing art, and, being such, must need the accompaniment of a thorough education to place it alongside of other professional vocations. Does the essayist think that, had Chapin A. Harris remained in his laboratory and at his chair, carefully hiding and cherishing the light of the lamp his bushel contained, totally ignoring the instincts his nature had developed, to "go teach all nations" what he had learned, that he could have benefited the profession and the public to any extent to be compared with the fruits of his self-sacrificing, industrious, all-inspiring, ambitious life? Does he think that the time and skill of our fully-appreciated Sudduth could have borne fruit of value and importance to the standing of our profession in any way comparable with its acknowledged worth, had he confined himself to the chair and the bench? And although not all of us, indeed, extremely few, can have our names inscribed upon the professional escutcheon alongside of Miller, of Allen, and of Atkinson, will he not be sufficiently magnanimous to confess our debt is due to their theory and their research, and not to their ability to make a gold plate or contour a defective molar? Dental education, he asserts, is to make dentists, and with this I have agreed. But let us not forget the new meaning advance has placed upon that word. Mechanics, nowadays, are not dentists, and no one gift is more essential and important to the aspirant for our degree, but I am convinced that while dentists may be produced by education, mechanics are born, not made. And what must be the result of this phenomenal advance and spread? Simply that the dental profession, following in the footsteps again of its brother medicine, must divide itself into branches of special practice. Taken in its entirety to-day, one head wishing to contain, to master, and to appreciate all that is implied by doctor of dental surgery must indeed be a paragon or a prodigy. Indebted as we are to the different sciences,—anatomy, physiology, chemistry, physics, materia medica, therapeutics, biology, mycology, etc.; called upon to advise and expected to treat almost any and every pathological condition hidden behind the orbicularis oris, does it strike you as consistent to believe that a smattering of a knowledge of any of our collateral sciences, such as might be gained by listening to a few short lectures prepared, culled, and condensed by our most eminent specialists, will fit the average candidate to occupy a foremost place before his clientele and the public? No; while dental colleges have multiplied to an undesirable extent, and while it is true that many of them are mills for grinding professional grist, simply and only as a secondary consideration, their first being financial greed and imaginary professional promotion and standing to its corps of professors, I must claim that from even this state of affairs has arisen an upward and an onward march towards our professional utopia paralleled by the strides and accomplishments of no other vocation. I am glad that the essayist has noticed and appreciated the value of this corrected system of education. I only regret the paradox of his conclusions, and fear the result upon the careless observers, and this, if any be necessary, I offer as my apology for what might at first glance appear as an attack or antagonism.

It appeared to me, while listening to the paper, that I could discern a tendency on the part of the writer to give little credit to, and place small importance upon, three important branches of the required course,—viz., materia medica, therapeutics, and pathology. I hope I do not do him an injustice in this conclusion, but if I am correct I also hope he will not misconstrue my motives in the great stress I wish to place upon their importance. I want to be placed upon record as avowing that to no branch of dental education are we so much indebted for our progress, and whatever standing we may have attained, as to those mentioned. What is it that enables the progressive dentist of to-day to bid defiance to that host of pathological conditions so often brought to his notice, and upon the eradication of which depends the saving of those priceless organs, in the past relegated to the forceps, but at present known to be essential to a proper performance of true physiological function? Certainly I do not believe it will be the verdict of our profession that the work so ably done in the past by Black, Gorgas, Harlan, Miller, and a host of co-workers in this field amounts to *nil.* To the results of pathology we certainly owe our present understanding of the conditions existing. To the results of therapeutics we certainly owe our ability to combat and overcome those conditions; and to the results of analytical and synthetical chemistry, as applied to the field of materia medica, we certainly have placed within our reach the agencies necessary for this advanced work. Then, taking the debts we owe to these branches collectively, recognizing that through their agency we hold the power I have suggested, who will doubt the importance to be placed upon these studies to still further promote a proper recognition of our professional standing? Truly, a *little* learning is a dangerous thing, and the idea advanced by the essayist, that a smattering of any scientific branch is all a practitioner needs to know, I feel honestly called upon to refute. It is just this slipshod method of teaching that has so often brought some institutions of learning into bad repute, no less than the consciousness on the part of the recent graduate, that his education has not been sufficient to enable him to cope with more searching and progressive *confrères.* No; I would hold and verily believe we cannot know too much. Especially is this so of any and all branches which go to make the sum total of those sciences which form the foundation of general medical practice. I do not believe the essayist will deny that even a chief in the culinary department of any hotel, an ordinary cook, whose special education in his art had been supplemented by a general knowledge of chemistry, would be in possession of secrets and powers entirely unknown to his ignorant associate. Would not the possession of this knowledge render his services more valuable to his employer? would it not have a tendency to throw a certain amount of respect around his calling and a value upon his time not to be hoped for by others? If such would be the case in so menial a position, does it not multiply many times in importance as it rises upon the scale of vocations until professional mission is reached? True education is defined as educing, leading, or drawing out the latent powers of an individual. Let us not forget, however, that while this definition is philosophic, a more specific sense would lead us to the fact that technical training is necessary to fit the scholar, or the student, for the occupation by which he desires not only to earn his daily bread, but to ornament society. This necessitates elementary schools for the multitude, secondary schools for a smaller number, and universities for the highly-favored few. We want to remember that education embraces not only the facts of instruction and good breeding, but that it comprehends the formation of mind, the regulation of the heart, and the establishment of principles. A want of education will always be to the injury, if not the ruin, of the sufferer. And a want of proper instruction will always unfit a man for the society of the cultivated.

I feel so enthused, gentlemen, upon this subject, that it seems to me this discussion might be indefinitely prolonged, and that, too, to the advantage of those who would in the slightest degree diminish or underrate the advantages of any amount of education. Jealous though I am of personal and individual reputation, not less so am I of the repute of the calling I have selected. Frame such laws and place such restrictions at the entrance of our doors as will give us an aggregate of educated, accomplished, and competent members, and soon the dental profession will be looked to as an equal of the most learned. I trust each and every member present will contribute his quota to this discussion. I ask the indulgence of the club and the forbearance of the essayist for the time I have consumed and for some of the extreme views I may have advanced, but I am of the opinion that in this audience I can hardly stand alone in my desire to give full and adequate importance to scientific education.

*Dr. E. S. Talbott.*—The essayist has portrayed the longings and ambition of both rich and poor young men who study dentistry for pecuniary gain.

It is not only excusable, but even the duty of a man to think of the support that a calling will yield him and those depending on him. He should therefore consider all before he decides upon entering a vocation that requires several years of preparation and considerable expenditure of money. A mistake made early in life may prove disastrous in the end and force him to try some other mode of making a living.

The student, having decided to make dentistry his life-work, selects his college and pays his fees. Up to this point he is responsible for his own actions, but as soon as he has secured his tickets the college is responsible in a measure for his success or defeat in life.

How great this responsibility! Does he receive a preliminary examination to test his ability to understand the lectures and pursue his studies intelligently? We have known students visiting this country, attending college, and returning to their homes without understanding simple English, and yet they took a diploma with them. We know of others that graduated lately and cannot read or spell correctly.

These are the criticisms made by the public.

To my mind there is another preliminary examination far more important to the student than this. I allude to the student's capacity for acquiring mechanical and manipulative skill. Without this no student can become successful, whatever his training may be otherwise. His ability should be tested, and only when found fair should he be admitted to the college with a view to graduating. The college having selected the proper material should have a uniform standard by which to gauge each student as he progresses from year to year. This can only be accomplished by a graded course. Uniform methods can be followed in dental colleges because dentistry is a positive art, unlike medicine and surgery, which still involve questions that are unsettled. With three courses of lectures in separate years the student should be qualified to pass a rigid examination. Mental training and manipulative skill are not enough to make an efficient dentist. The student should learn honesty in his business relations, cleanliness in his habits, and tact in handling patients, all of which are essential to success. At the age the average student enters college the mind and character are plastic, receiving impressions readily; he looks upon the lecturer as a model to copy.

Flaming advertisements in the daily papers, flattering notices of colleges and members of the faculties one or two columns in length, and notices to do dental work at cost, which students know to be untrue, all tend to demoralize him and lower his standard. Is it to be wondered at that our city is full of advertising dentists offering their services cheap to the public? Will not men following these practices injure the college from which they are graduated?

The essayist advocates that the student shall confine himself to such branches as have direct bearing on the practice of dentistry. This, though more practicable, is not conducive to the advancement of the profession. It is owing to the prevalence of this opinion that such statements as "the profession of dentistry is largely written out" are made in perfect good faith.

To this view the editor of the *Cosmos* answers by giving a long list of subjects for papers or books, all of which require preparation and study for which the student turned out in the way suggested above is unfitted. Should his ambition rise high enough to make observations and experiments, his efforts would be constantly frustrated by his ignorance of the studies that form the basis of these subjects and his lack of mental discipline.

There are two ways in which many of the existing evils can be corrected. The first is suggested by Dr. Ottolengui, of New York. He suggests the establishment of a National University. The student would attend a three years' course at the ordinary college and pass his examination. The college would give him a certificate which would entitle him to an examination at the university. The degree of Doctor of Oral Surgery is conferred upon him after having passed this examination. If he should fail he would be returned to his college for another course. This method would stimulate the colleges to do better work, for their existence would depend upon it. All students would graduate on the same basis.

A second way is the adoption of laws similar to those of Minnesota and Massachusetts, on the part of all States giving boards power not only to establish a standard for the different practitioners but also for the colleges. The State Boards should then appoint a National Board whose duty it shall be to examine into the workings of the colleges. This would force them to do better work when below the standard, for if they should fail to do this they would be ruled out. The National Board, not being a legalized body, could only act as an agent for the State Boards.

The State Boards, being legalized, could rule out such colleges as are not up to the standard. This is a mere suggestion of plans that might be pursued.

*Dr. W. W. Allport.*—There are many things in Dr. Smith's paper to commend, and with which I agree, but there are some ideas in it with which I cannot agree, and think it will do harm to let the paper be published and go forth in its entirety with the apparent sanction of all the members of the club. In addition to the criticisms that have already been made, I wish particularly to express myself as being opposed to the idea that we should have more of the practical, at the expense of the scientific, taught in our dental colleges, or that there should be relatively more of the practical than of the science of dentistry taught than we now have, for it is only upon the science that an intelligent practice of dental surgery can be pursued.

When I commenced the study of dentistry, with a few exceptions, the teaching was almost wholly practical. Dentists were not expected to understand anything except the manipulation necessary to clean, fill, and extract teeth, and put in sets of artificial teeth, and this was usually taught in private offices in about six months, there being but one dental college then in existence. Since then great improvements have been made in the instruments and materials used, as well as in manipulation, but it is the development of the science of our calling that marks the great difference between dentists of forty-five and fifty years ago and those who are now educated in accordance with the highest grade of modern teaching. Then the highest skill of the dentist was reckoned to be in the handicraft of the artisan; to-day his greatest skill has its foundation in his knowledge of the nature and scientific-medical treatment of dental diseases. Say what you will, the main object in learning any business is to make money at it, and, in most cases, when a young man enters upon business on his own account, his first and leading object is to this end. If a dentist has not acquired the habit of study, and become versed in the science of his calling while a student, the chances are that, when he enters practice, his thoughts and energies will be mainly directed to that which most readily brings money,—the "practical," the manipulative part, at the expense of the scientific, upon which only an intelligent practice can be based. But let a young man go forth fully equipped in the science, and with but a moderate amount of technical teaching or acquirements, the incentive of money-making will readily prompt him to make up the technique that he may have failed to learn while in college. By this let it not be understood that I would have the student learn less of the technique than is now being taught in our dental colleges, but rather that I would have more science, more of what might be called medicine, taught to dental students than most of them now get. If either is abridged, let it be the practical, for that, as I have before said, would be more apt to be made up in after life.

For the proper scientific teaching of dental students, I regard the joint teaching in medical colleges and dental schools far preferable to the teaching in what is known as independent dental colleges,—better calculated to make intelligent and broad-minded practitioners. It is a common remark among dentists that medical men know nothing, or are exceedingly ignorant of the diseases of the teeth, and that therefore it is a waste of much valuable time to attend medical lectures in a medical college; and it is a common remark for medical men to say that dentists know but very little of what medical men know; that dentistry is almost entirely mechanical, or manipulative. The unfortunate thing about these two statements is, they are both too true. The ordinary medical man knows but very little of the special pathology that should be possessed by every practitioner of dentistry, and yet he possesses that broad scientific, medical knowledge upon which all special pathology must be based, without which foundation no man can become even an intelligent *student* of a specialty, to say nothing of an intelligent practitioner of it. But let anything be said to an independent dental college-man about this broad medical teaching for a dental student, and his reply will be, There is no need for it, and besides that, we have medical men in our faculty to teach dental students all they need to know of the science of medicine; and they will make this statement in the face of the fact that they say physicians know very little about the diseases of the teeth, or what dentists should know, as well as that the physicians feel that dentists need but very little medical knowledge.

Between the fact that medical men possess but little of the special knowledge that dentists should understand and the fact that medical men think that dentists need but little medical knowledge, it is evident that the dental student gets but a very limited amount of the latter by the teaching of medical men in these independent dental colleges, for they certainly would not teach them more than they feel or think they need.

To illustrate what is meant: Within the last three years, I happened to be present at a lecture upon the physiology of digestion and assimilation, by a medical man in one of these colleges. After it was over, occasion was taken to compliment him on his lecture, to which he replied, "It was not very full, or in detail what I would have given to a medical class." "Why," said I, "should not a dentist have just as perfect a knowledge of the physiology of digestion as a regular physician?" "Oh," said he, "it would do no harm, but it is not usually supposed to be necessary." At a later time I was in another dental college when a physician was giving a lecture on sarcomatous growths. In talking to him after the lecture was over, he said, "I hope you were interested in my lecture, but," said he, "it would have been very different had I been delivering it to a class of medical students." "Why so?" said I; "such growths are not infrequent in the mouth, and dentists should certainly be able to diagnosticate them when they see them, if not to operate upon them, when operations are indicated." "Oh," said he, "we have surgeons for that purpose, and it is not necessary for dentists to know much about such matters." And this is about the extent of medical teaching that dentists usually get in this class of colleges from medical men.

It is just as important that the dentist be well grounded in the fundamental sciences that go to make up a medical education as it is that the oculists or the gynæcologists should be, and in no place are these as well taught as in medical colleges, except in the university system of teaching; but a dental department in a university, where its teaching is all independent of the scientific departments of those universities, is in no way any better than the ordinary independent dental college, for they derive no benefit from the well-known State university system of teaching. If I could have my way, no student should be graduated in dentistry until after he had received a general medical education; but next to this he should receive jointly with medical students all that goes to make up the fundamentals of a medical education. He should recite from the same books, attend the same lectures, and submit to the same examinations in anatomy, physiology, chemistry, materia medica, and therapeutics, as well as in the general principles of pathology and surgery, as do those who expect to graduate in medicine, and then take up the practice of any of its specialties, pursuing his special studies in dentistry in a department devoted to that purpose, and nothing less than this will ever make the dental practitioner as intelligent in the treatment of the diseases that come within the scope of his practice as are those who are specialists in the treatment of other diseases.

But, say those who are opposed to this thorough teaching, why is it necessary for a dentist to understand the size of the pelvis, or of the surgery of parts remote from the mouth ? As well might it be asked, Why should the oculist understand the size of the pelvis, or the bones of the leg and feet ? and yet, who would want to employ an oculist who had not this general medical intelligence ? And there is not a principle in surgical pathology or therapeutics but what is applicable in the treatment of the diseases that come within the scope of the practice of the true dental and oral surgeon. And without a knowledge of these principles, supplemented by necessary special knowledge, he cannot meet the highest requirements of his calling. Instead of the scientific or medical portion of a dental student's education being doled out to him in such stinted portions as medical teachers in dental colleges think he needs, it would be far better for him to occupy his time in the study, and enjoy the broader teaching in his medical studies that is laid down and exacted in our best medical colleges, that he may pass without favor the same examinations by the chairs that is required of those who expect to take the medical degree, as is now required by some, at least, of these joint schools,—the dental students scorning to ask or receive any favors in their medical examinations. A dentist thus grounded in his knowledge of the principles of general medicine would himself be able to prescribe such remedies in the treatment of diseases, and make use of such means as his judgment might suggest to him, as being best adapted to the case in hand, as other specialists do, rather than to shape his treatment in accordance with the very limited medical teaching of men who have but a faint conception of the requirements essential to the proper treatment of dental diseases.

*Dr. C. F. Hartt.*—A middle course is generally the safest. A well-rounded dentist is a safe man, and should be reasonably well educated. As much depends on the student as on the college. If there is a hungering and thirsting for knowledge, colleges will afford the means of supplying the want.

*Dr. C. N. Johnson.*—I am heartily in accord with the spirit of the paper, and also agree with much that has been said in apparent criticism of it. In fact, I think the two sides are not so far apart in belief as would appear, and that some things said in the discussion are due to a misconception of the paper. I do not believe that the essayist meant to maintain that nothing but practical branches shall be taught. The argument of the paper, as I understand it, was that in the time given for studentship it was not possible for the average student to gain a thorough scientific knowledge and also a perfect practical training, and that where it was obviously necessary to slight something, it was better to lessen the theory and devote more time to practice. This I consider was a good argument. A student armed only with scientific knowledge is not a proper person to send out to practise dentistry. The principal fault with dental education to-day is that we do not get the right kind of students into the colleges to begin with. It is impossible to pour a quart of material into a pint measure and make it stay there. Our methods of matriculation are too lax. If we, as a profession, are to make an impression on the world, we must be intellectual men, and we cannot, in a short course in the dental college, gain sufficient knowledge to entitle us to that distinction. A man should be educated before he enters a college,—he should know *how* to study. A young man just from the plough with mind untrained loses half his first term before he can begin to learn anything. A uniform standard of requirements for matriculation should be adopted and adhered to by all colleges. It is too manifest that students without an education are often allowed to matriculate on the theory that if one college does not admit them another will.

In Canada the law governing applicants is far in advance of present methods here. The college holds no matriculation examination whatever, but requires the applicant to show at least a provincial third-class teacher's certificate, including a knowledge of Latin, and in Canada this means something. Examinations are held with great strictness, and the applicant who presents such a certificate has at least the foundation for a good education. This question of matriculation should be carefully considered, and concerted action taken on it by all our colleges.

*Dr. E. M. S. Fernandez.*—One thing that would be a great improvement in dental teaching is that the student should know something about drawing. Drawing should be used in every effort to teach. He would require lineal and mechanical drawing to be included in a preliminary examination. Another improvement needed is that the professors should be made such by some authority. Professors are now self-styled as such. We need a place to teach men how to teach.

*Dr. E. J. Perry.*—With some noble exceptions, colleges have degenerated into a *business;* the commercial idea has crowded out the idea of teaching. Colleges profess to teach ethics, and in the infirmary they teach the most improved methods of quackery. Professorships have grown cheap. The commercial idea in colleges prevents the proper teaching as to the construction of metal plates. This idea is ruining the colleges. Colleges graduate men and send them out to compete with the infirmaries of the same institution. Think of a theological seminary organized for profit, or of any educational institution run for money!

*Dr. C. R. Baker.*—The idea of profit was not first established or practised in colleges. Students were taken into private offices for profit, and the mistake was in asking them two hundred or three hundred dollars for what they would get there, which would not be one-tenth of what he would give. Students were taken in for a profit, and how much were they taught? It is a mistake to advise students to go first to a dental college. They should first go into a private office. The first thing they should see is the refinement of a private office; in a college only slip-shod methods obtain, and the object is to get in as many fillings as possible. If they first go to a college, they know nothing of the terms used. They may go to a university with students who have studied for some months, yet they are expected to know as much as they do. They take lectures the same as the medical student, but they are cut off from laboratory or hospital practice. He regrets every day that he knows so little about drugs and therapeutics. It is impossible to know too much of science, yet we want all the practical knowledge we can get. We want to get at principles, yet we cannot go all through the materia medica. To lay a foundation is all we can do; but what we do take up should be thoroughly mastered; go to the bottom.

*Dr. J. H. Woolley.*—The ethical side of dental education will have its influence in the profession at large. When the discussions on these subjects are read, more thought on them will be aroused.

*Dr. A. E. Baldwin.*—It is probable that the two sides of this discussion are not so far apart as they appear. A profession is adopted from self-love and self-interest: therefore, when beginning it, the person should be competent, and not learn it afterwards. First, the dental student should be capable of understanding the principles brought before him. Honesty should be taught him. He is now taught lies from the day he enters a college, not *as* lies, but as profits. Dental work is advertised to be done "at cost." A student asked the cost of an oxyphosphate filling, which was about one cent, but when put in by a professor "at cost," the amount was three dollars and seventy-five cents. Professional life is begun in iniquity. Can a student put confidence in such professors? On the contrary, he looks with suspicion on the man who deceives him. The students do as do the professors. The preliminary examination should be thorough and should be insisted on. If a dental college is properly arranged and officered by those who have ostracized profit, there is no place where a student may so well begin. The first impressions are lasting ones; it is easier to learn right than to unlearn. It is impossible to teach all subjects thoroughly; the elements should be fully taught, and the rest may be elaborated afterwards. Half the task is accomplished when the student has learned how to study. When I first entered a dental college they had no demonstrator at all. There are not demonstrators enough in the colleges. Some students require a demonstrator all to themselves. The medical profession is spoken of in raillery by dentists, but it is never the case that medical men speak so about dentists. Medical men are lamentably ignorant of "gum-biles," but they are glad to have their attention called to anything that helps them. We are apt to glorify ourselves too much. If we realized how little we know, we would study more.

*Dr. A. J. Nichols.*—If we are members of a profession, why should we not qualify as general practitioners? We treat too much locally; we do not look beyond a filling. What per cent. of the dental profession is capable of prescribing for a patient. A thorough medical course should be required in a dental college. A student should go to a private office first.

*Dr. C. S. Smith,* in closing the discussion, said: It is plain that the real purport of the paper has been misapprehended, as was perhaps to have been expected. It was not the intention to belittle education; but quite the contrary. What is contended for is that if the amount must be limited, what is obtained should be of the right kind. In all the talk about the necessity of a complete medical education for dentists, the fact is lost sight of that the object of dentistry is to save teeth,—and from what? From dental caries, which it is not at all certain is a *disease;* the treatment of it, when simple, is, aside from sensitive dentine, purely mechanical. We talk of educating the people up to an appreciation of their teeth, and taking proper care of them; if the day ever comes when this is done, there will be no need for any medical knowledge whatever, aside from oral surgery and the treatment of irregularities, for dexterous manipulation will then be about the only thing required for success.

*Monday, April 28, 1890.—Evening Session.*

The Chicago Dental Club met as usual, and the evening, aside from routine business, was mainly consumed in hearing from Dr. J. N. Crouse, who had been given the floor to explain the objects of the Dental Protective Union, and in remarks by members on this subject.

At the close of the discussion the following resolution was unanimously carried:

"*Resolved,* That the Chicago Dental Club endorse the Dental Protective Association as being worthy of the confidence and support of the profession."

Adjourned.

C. Stoddard Smith, *Secretary.*

